# VETERAN- AND CAREGIVER-REPORTED FACTORS IMPACTING VETERAN-DIRECTED CARE (VDC) PROGRAM ENROLLMENT AND EXPANSION

**DOI:** 10.1093/geroni/igae098.3514

**Published:** 2024-12-31

**Authors:** Wendy Hathaway, Pranjal Tyagi, Erin Bouldin, Samer Nasr, Stuti Dang

**Affiliations:** VA Providence Healthcare System, Tampa, Florida, United States; South Florida Veteran Affairs Foundation for Research and Education (SFVAFRE), Miami, Florida, United States; University of Utah, Salt Lake City, Utah, United States; Veterans Integrated Service Network (VISN) 8., St. Petersburg, Florida, United States; Miami VA Healthcare System, Miami, Florida, United States

## Abstract

The Department of Veterans Affairs (VA) is increasing access to Home- and Community-Based Services (HCBS) as an alternative to long-term institutional care (LTIC). Veteran Directed Care (VDC) is a self-directed HCBS program that empowers veterans at risk of LTIC to hire their own care providers. The VA is increasing VDC access by expanding the program to all VA medical centers (VAMCs) nationally. As part of a multi-aim and mixed-method evaluation project examining barriers and facilitators to expanding VDC, qualitative semi-structured interviews were conducted across seven VAMCs in Florida, South Georgia, and Puerto Rico with two groups of veterans who were referred to VDC: those that enrolled in the program (n=2) and those that declined (n=2). We also interviewed family caregivers of veterans in both groups (n=2 each). Interviews were conducted via Microsoft Teams from January-March 2024 and recorded with auto-transcription. Data were analyzed using rapid qualitative analysis techniques. Enrolled veterans and their caregivers pointed to several facilitators including the ease of enrollment; user-friendly management tools; compensation for care; role recognition; the ability to choose their caregiver and schedule; the reliability and trustworthiness of self-selected care providers; and relationships formed. Those who declined to enroll identified several barriers such as the program complexity; burdensome employee management tasks; fear of losing eligibility for other programs and services; and feeling the compensation was inadequate. Interviews are still ongoing. Understanding the veteran and caregiver perspectives on enrollment will inform strategies and mechanisms to support VA’s goal of increasing VDC access and veteran participation.

